# Spatial Balance of Photogenerated Charge Carriers in Active Layers of Polymer Solar Cells

**DOI:** 10.3390/molecules28155823

**Published:** 2023-08-02

**Authors:** Chan Im, Sang Woong Kang, Jeong Yoon Choi, Jongdeok An, Júlia Mičová, Zdeněk Remeš

**Affiliations:** 1 Department of Chemistry, Konkuk University, 120 Neungdong-ro, Gwangjin-gu, Seoul 05029, Republic of Korea; tony22@konkuk.ac.kr (S.W.K.); kirisi@konkuk.ac.kr (J.Y.C.); anhijuk@konkuk.ac.kr (J.A.); 2 Institute of Chemistry, Slovak Academy of Sciences, Dúbravská cesta 9, 845 38 Bratislava, Slovakia; chemjumi@savba.sk; 3 Institute of Physics, Czech Academy of Sciences, Na Slovance 1999/2, 182 21 Prague 8, Czech Republic; remes@fzu.cz

**Keywords:** polymeric photovoltaics, non-fullerene acceptors, internal absorption, internal quantum efficiency, transfer matrix method, spatial balance, charge carrier distribution

## Abstract

Bulk heterojunction polymer solar cells (PSCs) blended with non-fullerene-type acceptors (NFAs) possess good solar power conversion efficiency and compatibility with flexible electronics, rendering them good candidates for mobile photovoltaic applications. However, their internal absorption performance and mechanism are yet to be fully elucidated because of their complicated interference effect caused by their multilayer device structure. The transfer matrix method (TMM) is ideal for analyzing complex optical electric fields by considering multilayer interference effects. In this study, an active layer (AL) thickness-dependent TMM is used to obtain accurate information on the photon-capturing mechanisms of NFA-based PSCs for comparison with experimental results. Devices with AL thicknesses of 40–350 nm were prepared, and the AL-thickness-dependent device parameters with incident photon-to-current efficiency spectra were compared with the calculated internal absorption spectra of the TMM. The spectrally and spatially resolved spectra as a function of the AL thickness and excitation wavelength revealed that the power conversion efficiency of the NFA-blended PSC decreased with the increasing AL thickness after reaching a maximum of ~100 nm; by contrast, the internal absorption efficiency showed the opposite trend. Furthermore, the TMM spectra indicated that the spatial distribution of the photogenerated charge carriers became significantly imbalanced as the AL thickness increased, implying that the AL-dependent loss stemmed from the discrepancy between the absorption and the extracted charge carriers.

## 1. Introduction

Non-fullerene-type acceptor (NFA)-blended polymer solar cells (PSCs) have attracted immense interest owing to their applicability in solar power conversion efficiency (PCE) [1,2,3] together with mechanical flexibility [4,5] and solution processability [6]. In addition to these promising properties, PSCs have an additional substantial advantage because of the relatively high optical density of their active layers (ALs) compared to conventional silicon solar cells, which enables the preparation of material-saving thin ALs.

However, conventional bulk heterojunction (BHJ)-type PSCs must be prepared with multilayered stacking structures to achieve optimal PCE. This can be explained by the properties of the BHJ AL, which include low electrical conductivity and less compatibility with inorganic and metallic materials. Therefore, the PSC requires not only an AL, but also subsidiary buffer layers with electrodes, such as a transparent conducting layer and a top opaque metallic contact layer. Well-known examples of buffer layer combinations include poly(3,4-ethylenedioxythiophene):poly(styrenesulfonate) (PEDOT:PSS) and lithium fluoride (LiF) for forward PSCs, and zinc oxide (ZnO) and molybdenum oxide (MoO_3_) for inverted PSCs. Nonetheless, most layers have thicknesses ranging from several to tens of nanometers [7]. Such multilayer structures with ALs and a reflective opaque metal electrode are ideal for enhancing the interference of light traveling inside PSCs [8,9].

The PCE of a PSC is expressed as the ratio between the input solar power (P_in_) and the output electric power (P_out_), as shown in Equation (1). P_out_ can be calculated by multiplying the short-circuit current density (J_SC_), open-circuit voltage (V_OC_), and fill factor (FF), which can be extracted from a current density–voltage (J-V) measurement using a known P_in_.

(1) $$\mathrm{PCE}=\frac{P_{\mathrm{out}}}{P_{\mathrm{in}}}=\frac{\;J_{\mathrm{SC}}\cdot V_{\mathrm{OC}}\cdot \mathrm{FF}}{P_{\mathrm{in}}}$$

Among the device parameters, the J_SC_ has a distinctive meaning for the relationship between the number of incident photons and the number of captured photons, which can be further converted to charge carriers (CCs) in a quantum manner, that is, one photon to one CC is typical for such single-photon devices. Therefore, J_SC_ measured at 0 V bias is often regarded as a measure of the CC photogeneration efficiency of a photovoltaic device. The quantum-wise conversion efficiency from incident photons to detectable currents over various processes must be bound with inadvertent losses. Therefore, a careful analysis of the difference between the J_SC_ and extracted CC enables us to understand the various loss processes in detail, which is essential for improving the knowledge and performance of PSCs [10].

The overall extracted CC photogeneration efficiency from the viewpoint of the J_SC_ (η_total_) can be expressed as a collective multiplication of the various underlying processes. A simple example is presented in Equation (2) for a better understanding, although the processes involved in practical PSC operation are more complex. The most important initial process of a PSC is photon absorption by the AL, followed by instantaneous primary exciton formation in the AL (η_Phabs_) [11]. As a next step, the generation of free CCs from captured photons (η_CCgen_) can be considered. Subsequently, the photogenerated CCs, electrons, and holes are transported toward each electrode (η_CCtr_) and extracted to the external circuitry (η_CCext_) for detection [12].

(2) $$\eta_{\mathrm{total}}=\eta_{\mathrm{Phabs}}\cdot \eta_{\mathrm{CCgen}}\cdot \eta_{\mathrm{CCtr}}\cdot \eta_{\mathrm{CCext}}$$

A further analysis based on Equation (2) can be performed in addition to the conventional J-V characterization of PSCs. Obviously, accurate information about the total incident light amount on a device and the absorbed light amount in an AL in a device is crucial because the ratio between these numbers is the determining factor for the initial step and acts as a criterion for the subsequent steps to evaluate the PSC performance in detail. This ratio is often expressed as the internal absorption (Q), as shown in Equation (3), and the Q of an AL is denoted as Q_AL_ in this study. The Q_AL_ can be combined with the incident photon-to-current efficiency (IPCE) in Equation (4) to form the internal quantum efficiency (IQE), as shown in Equation (5). The IPCE, often denoted as the external quantum efficiency (EQE), is conceptually the same as the η_total_ and can be used to calculate the J_SC_ with the proper assumption of P_in_, which can be compared with the J_SC_ extracted from the J-V characteristics.

(3) $$Q_{\mathrm{AL}}\left(\lambda \right)=\frac{\#\;\mathrm{of}\;\mathrm{absorbed}\;\mathrm{photons}\;\mathrm{in}\;\mathrm{an}\;\mathrm{AL}\left(\lambda \right)}{\#\;\mathrm{of}\;\mathrm{total}\;\mathrm{incident}\;\mathrm{photons}\left(\lambda \right)}\;$$

(4) $$\mathrm{IPCE}\left(\lambda \right)=\frac{\#\;\mathrm{of}\;\mathrm{extracted}\;\mathrm{CCs}\left(\lambda \right)}{\#\;\mathrm{of}\;\mathrm{total}\;\mathrm{incident}\;\mathrm{photons}\left(\lambda \right)}$$

(5) $$\mathrm{IQE}\left(\lambda \right)=\frac{\#\;\mathrm{of}\;\mathrm{extracted}\;\mathrm{CCs}\left(\lambda \right)}{\#\;\mathrm{of}\;\mathrm{absorbed}\;\mathrm{photons}\;\mathrm{in}\;\mathrm{AL}\left(\lambda \right)}=\frac{\mathrm{IPCE}\left(\lambda \right)}{Q_{\mathrm{AL}}\left(\lambda \right)}$$

Unfortunately, there is no straightforward method to measure the Q_AL_ directly. Conventional UV-visible (UV-VIS) absorption spectroscopy with an AL thin film on an incoherent substrate cannot be used to accurately estimate the Q_AL_ of a device because of the complex interference in multilayer PSC [9,13]. Therefore, it is necessary to calculate the Q_AL_ using, for example, the transfer matrix method (TMM). In addition, an AL-thickness-dependent TMM investigation can be performed to obtain more insight into the PSCs’ internal absorption as a function of the AL thickness, which can be used to understand and determine the optimum structure of a PSC with various AL thicknesses. The challenges in increasing the AL thickness not only include increasing the PCE due to the increasing photon capturing facility upon increasing the AL thickness, but also obtaining greater tolerance regarding the AL thickness for printed electronics to overcome the thickness limit of approximately 100 nm [14,15].

Although there are thousands of studies on the use of NFA-based PSCs to obtain higher PCEs and/or to understand the various underlying mechanisms therein, it is still difficult to find comprehensive studies on the TMM-mediated internal absorptions of such BHJ AL systems consisting of promising NFAs and their suitable polymer counterparts, although the TMM has already been well established for at least a decade [8,11,16] and has been successfully applied to fullerene derivatives [17,18]. This is undesirable when systematic AL- thickness-dependent TMM studies for NFA-based systems are considered, although there are well-formulated AL- thickness-dependent investigations [19,20,21].

Therefore, we investigated an NFA-blended PSC system via TMM analysis for a wide range of AL thicknesses from approximately 50 to 500 nm. To compare the TMM results with the practical device parameters and incident photon-to-current efficiency (IPCE) spectra of the PSCs, various PSCs with a wide range of AL thicknesses, from 40 to 350 nm, were prepared and experimentally characterized. Subsequently, the calculated internal optical situation considering the interference effect, the Q_AL_ spectra, and the calculated J_SC_ obtained via TMM, and the device performance and the J_SC_ extracted from the J-V measurements and experimental IPCE data, were comparatively analyzed. As expected, the results reveal that the experimental J_SC_ decreased after passing the PCE maximum formed near an AL thickness of 100 nm, although the number of photons captured increased with the increasing AL thickness. This means that the probability of the loss of photogenerated charge carriers (CCs) must increase with the increasing AL thickness. This is an important limiting factor for varying the AL thickness while maintaining the applicable PCE value.

A further TMM analysis of the spectrally and spatially resolved internal absorption of PSCs with various AL thicknesses indicated that the instantaneous spatial distribution of photogenerated CCs depended quantitatively on the AL thickness. It was found that the complicated distribution information could be simplified as a simple spatial balance factor by dividing the entire area into two sectors, front and rear areas, with a 50:50 ratio. It is noteworthy that the balancing factor and loss probability exhibit a reasonable correlation, which can be used to explain the CC loss probability as a function of the AL thickness.

## 2. Results

### 2.1. Thickness-Dependent PSC Properties

**Figure 1:**
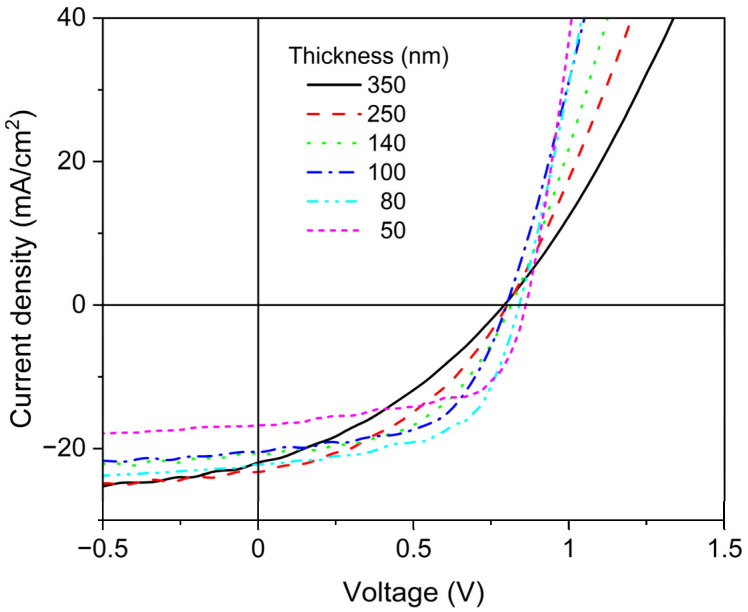
Current density–voltage (J-V) curves obtained with thickness- dependent PCE measurements.

Figure 1 shows the representative J-V curves of the selected PSCs with various active layer (AL) thicknesses ranging from 50 to 350 nm. The stiffness of the rising parts and the J_SC_ decreased with the increasing AL thickness. Consequently, the FF decreased with the increasing AL thickness. The device parameters extracted from the J-V curves of the selected devices in Figure 1 are listed in Table 1.

**Figure 2:**
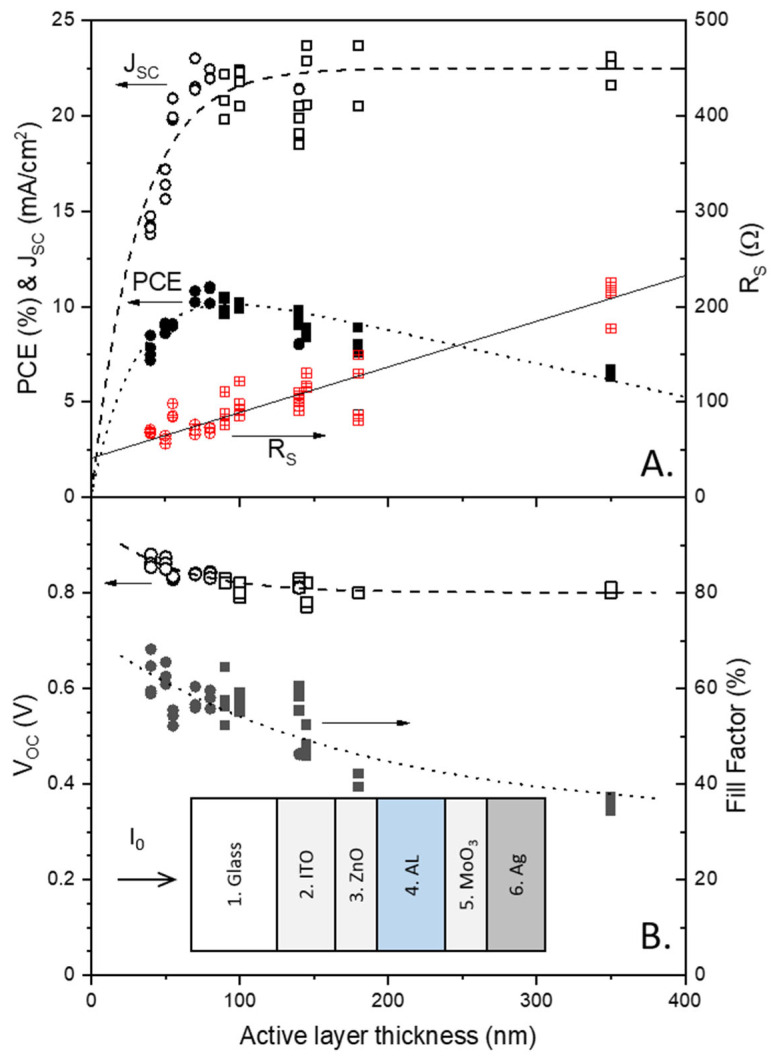
(**A**) Plots of J_SC_ (open symbols), PCE (solid symbols), and R_S_ (open symbols with cross characters); (**B**) V_OC_ (open symbols) and FF (solid symbols) as functions of the AL thickness. For both (**A**,**B**), results estimated with the lower concentration solution are plotted with circles, while those estimated with the higher concentration solution are plotted with rectangles. Inset: (**B**) Scheme of the studied device structure with the layer numbering convention used in this study.

In Figure 2A,B, all the parameters of the studied devices are plotted as functions of the AL thickness. As shown by the J-V curves in Figure 1, the FFs in Figure 2B decreased by approximately 50% with the increasing AL thickness when the initial thin AL thickness was compared with the final thick AL thickness. The V_OCs_ also decreased exponentially with the increasing AL thickness; however, the decreasing tendency became significantly moderate as the values decreased by only approximately 7%. The decreasing tendency of the FF is correlated with the increasing tendency of the series resistance (R_S_), as shown in Figure 2A. It is known from the diode equation formalism that the FF decreases when the R_S_ is increased, or when the shunt resistance (R_Sh_) is decreased. Indeed, the R_S_ increased with the increasing AL thickness, as the rising parts of the J-V curves flattened with the increasing AL thickness, as shown in Figure 1. However, the R_Sh_ was not affected by the AL thickness, as the horizontal parts of the J-V curves remained virtually the same over all AL thickness ranges. In the case of the thickest sample with an AL thickness of 350 nm, the slope of the J–V’s horizontal part increased, but this may be attributed to the decrease in the stiffness of the rising part being correlated with the R_S._ The AL- thickness-dependent V_OC_ could be explained with the built-in potential of the PSCs in addition to the concentration of space charge in the AL bulk presented by previous insightful studies [22,23].

While the V_OC_ and FF decreased with the increasing AL thickness, the J_SC_ increased with the increasing AL thickness. The increasing tendency of the J_SC_ as a function of the AL thickness is depicted with a dashed line in Figure 2A using Equation (6), where d, A, and B represent the AL thickness, the maximum J_SC_, and the slope of the exponential function, respectively.

(6) $$J_{\mathrm{SC}}\left(d\right)=A\cdot \left(1-e^{-\frac{d}{B}}\right)$$

**Figure 3:**
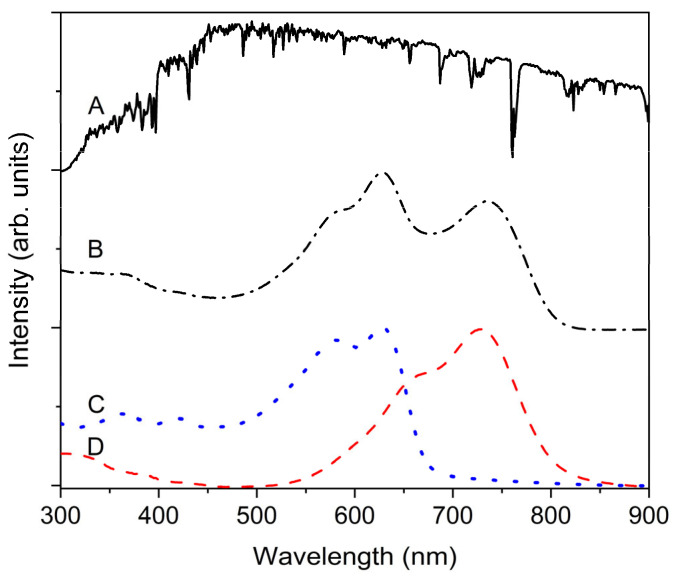
(**A**) Spectral irradiance spectrum at air mass of 1.5 G (solid line), (**B**) UV-VIS absorption spectra of AL film (dashed–dotted line), (**C**) PBDB-T-2F pristine film (dotted line), and (**D**) ITIC-4F pristine film (dashed line).

For the calculated dashed line shown in Figure 2A, the values of 22.5 and 130 were used for A and B, respectively. Notably, d was multiplied by a factor of 1.8 for the above calculation, which followed the incoherent Beer–Lambert law. The factor of 1.8 is reasonable because the incident light travels through the AL twice owing to the reflective metal electrode. Fitting with Equation (6) implies that the J_SC_ must be proportional to the number of photons absorbed by the AL, where most charge carriers can be generated. To analyze the photon-capturing ability of the AL, the conventional absorption spectra of a single-layer sample, as presented in Figure 3, with the incoherent Beer–Lambert framework, can be used if there is negligible interference. For practical calculations, such as J_SC_ prediction, the total amount of incident light can be regarded as the spectral irradiance [24], as shown in Figure 3.

The PCE increases with the increasing AL thickness until the AL thickness reaches approximately 90 nm, as shown in Figure 2. After reaching a maximum near 90 nm, the PCE decreases linearly with the increasing AL thickness. This can be explained using Equation (1), where the numerator P_out_ is calculated by multiplying J_SC_, V_OC_, and FF. With the increasing AL thickness, the J_SC_ increased but gradually saturated, whereas the FF decreased. Consequently, their product PCE can form a maximum PCE near an AL thickness of 90 nm and then decrease virtually linearly with the increasing AL thickness. This feature is comparable to that reported in the literature on the thickness dependency of organic solar cells [20,21].

The AL thickness dependence of the PCE can be quantitatively but rapidly understood by the fact that photon absorption increases with the increasing AL thickness in conjunction with at least one loss process, which increases linearly with the increasing AL thickness. Evaluating the relationship between photon absorption and loss as a function of the AL thickness is crucial for understanding and improving PSCs. However, interference due to the multilayer device structure of PSCs is a hurdle in estimating their internal absorption with acceptable precision. Indeed, their interference effect can be recognized from the waviness of the J_SC_ curve, as shown in Figure 2A, in addition to the deviation between the UV-VIS spectra in Figure 3 and the IPCE spectra, which will be shown later in this study.

### 2.2. TMM Analysis of Photon Absorption in an AL

**Figure 4:**
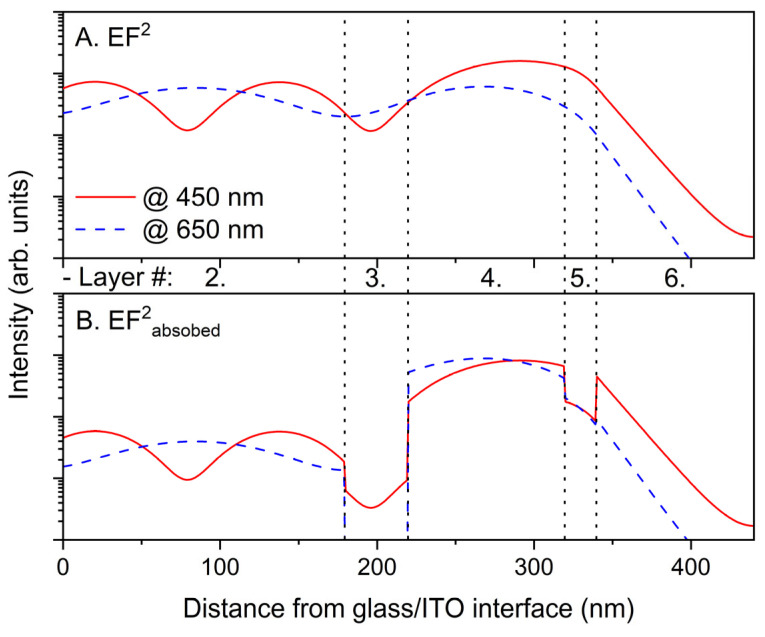
(**A**) EF^2^ of penetrated light into a device. (**B**) Absorbed intensities from EF^2^ in a device; traces at 650 nm (solid line) and 450 nm (dashed line) of a device with a 100 nm AL thickness.

To estimate the internal absorption of the AL (Q_AL_) in an OPV device, the TMM can be applied with the optical constants of all layers, as described by Pettersson et al. [8]. In Figure 4, some representative results of the TMM calculation of a PSC device with a 100 nm AL thickness are shown. In Figure 4A, light intensities at wavelengths of 450 and 650 nm as functions of position in the incident light direction are shown as squares of the electric field amplitudes (EF^2^). In Figure 4B, the absorbed amounts of the light intensities from the EF^2^ are also shown for wavelengths of 450 and 650 nm. The interfaces between the layers are indicated by dotted lines. Between Figure 4A,B, specific layer numbers are displayed as well.

For a complete calculation via the TMM, all the optical constants of the involved layers, including a thick glass substrate, must be considered. However, millimeter-scale glass substrates cannot be treated as other layers owing to their incoherent nature. Therefore, the thick glass substrate was treated as an incoherent layer, for which no explicit interference effects were considered [16]. Therefore, all the layers involved are shown in Figure 4, except for the incoherent glass substrate, although its conventional reflectance, transmittance, and absorptance spectra are shown in Figure S3 of the Supplementary Materials for reference purposes. It can be clearly seen that the square of the electric field, that is, the light intensity in a device, has a wavelength-dependent continuous shape in conjunction with interference features that are intensively affected by the well-reflective opaque metal electrode (layer 6). After obtaining the light intensity in a device via the TMM, the absorbed portion of the light intensity can also be calculated, which becomes a criterion for the internal absorption of each layer in a spectrally and spatially resolved manner. Indeed, the TMM has been a well-established methodology to model optical situations for such multilayer PSC structures since Pettersson et al. presented their study [8] and used various groups to analyze the internal absorption of various types of solar cells [25,26]. Therefore, we did not describe the fundamental aspects of the TMM in more detail in this study, except for some specific aspects.

**Figure 5:**
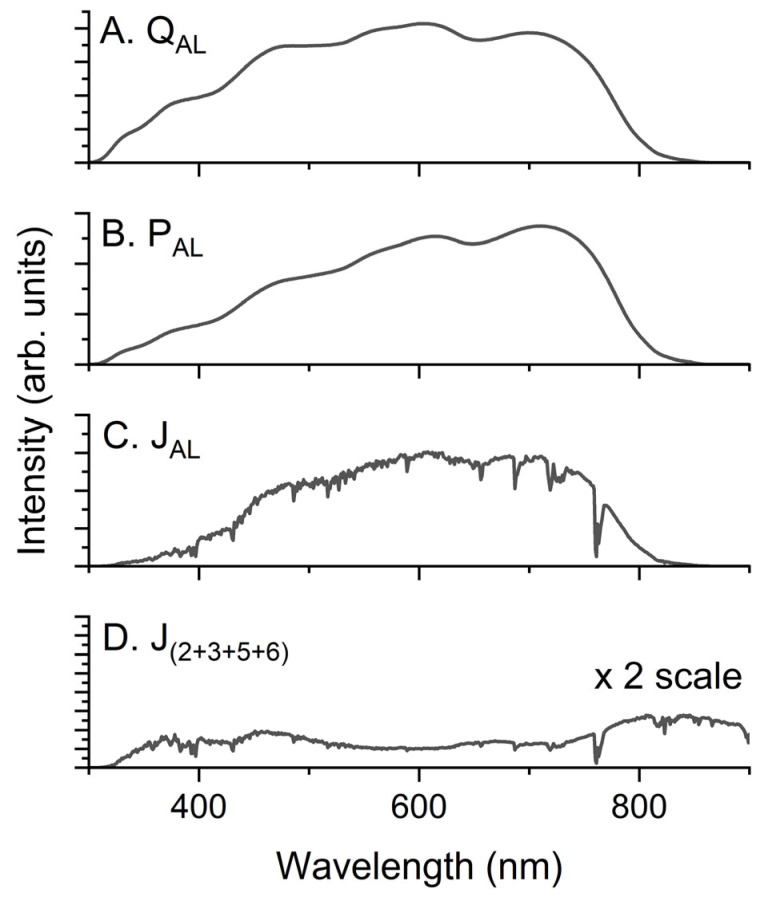
(**A**) Q_AL_, (**B**) P_AL_, (**C**) J_AL_, and (**D**) J_(2+3+5+6)_ for a 100 nm thick AL.

Subsequently, the EF^2^_absorbed_ can be spatially integrated over the entire distance, that is, the full thickness range, of an AL to form the internal absorption of the AL (Q_AL_), as expressed in Equation (7). An example Q_AL_ spectrum is shown in Figure 5. Notably, the internal absorption spectra can be calculated for any position or range of the device included in the TMM calculation.

(7) $$Q\left(\lambda \right)=\int_{\mathrm{start}\;\mathrm{position}}^{\mathrm{end}\;\mathrm{position}\;}{\mathrm{EF}}_{\mathrm{absorbed}}^{2}\left(\lambda \right)\cdot d\lambda$$

As soon as the Q_AL_ is obtained, the J_SC_ can be calculated by assuming that the CCs are directly generated from the primary excitons with a certain conversion efficiency. This conversion efficiency can be compared with the η_CCgen_ in Equation (2). Through this conversion, exciton diffusion-controlled and/or monomolecular- recombination-induced losses can occur. However, those losses are not explicitly included in the TMM-aided J_SC_ calculation because many research groups support that the CC photogeneration is very effective [26], and monomolecular recombination is also by far depressed in such a well- optimized BHJ blend system [27]. These are also the reasons why spatial balancing must be a thickness-dependent loss process, which will be addressed later in this study.

To calculate the practical number of photogenerated CCs, the incident light on a pixel of a PV device must be estimated to compare with the experimental J_SC_ using J-V measurements. This can be obtained by means of spectrally resolved light power measurements, resulting in the incident light power density H(λ) (W/m^2^). The estimated H(λ) can be converted to the photon flux density Φ(λ) (s^−1^ m^−2^) according to Equation (8). Because the photon flux density is the total number of incident photons per unit time and area, this value can be directly related to the number of photogenerated CCs per unit time and area. In Equation (8), q, h, and c represent the elementary charge, Planck constant, and speed of light in vacuum, respectively.

(8) $$H\left(\lambda \right)=\Phi \left(\lambda \right)\cdot \frac{h\cdot c}{\lambda \;\left(\mathrm{nm}\right)}=\Phi \left(\lambda \right)\cdot q\cdot \frac{\;1239.8}{\lambda \;\left(\mathrm{nm}\right)}$$

For practical purposes, for example, a comparison between various systems, H(λ), can be obtained from the standardized spectral irradiance, S_Irr_(λ), with a typical unit of W/nm·m^2^. An example spectrum is shown in Figure 3A. Consequently, Φ(λ) can be calculated using S_Irr_(λ) by following Equation (9). Notably, Δλ in S_Irr_(λ) represents the spectral interval with which each data point of S_Irr_(λ) is estimated and delivered. Using Equation (10), J_SC_(λ) can be calculated with either the experimentally estimated or TMM-calculated efficiency, IPCE(λ), with the standardized P_in_, S_Irr_(λ). Finally, the spectrally resolved J_SC_(λ) can be integrated over the entire spectral area of interest, from 300 to 900 nm, for the NFA-based AL, as expressed in Equation (11). This integrated J_SC_ can be compared with the conventional J_SC_ extracted from the J-V measurements.

(9) $$S_{\mathrm{Irr}}\left(\lambda \right)=\left(\Phi \left(\lambda \right)\cdot q\cdot \frac{\;1239.8}{\lambda \;\left(\mathrm{nm}\right)}\right)/∆\lambda =H\left(\lambda \right)/∆\lambda$$

(10) $$J_{\mathrm{SC}}\left(\lambda \right)=\mathrm{IPCE}\left(\lambda \right)\cdot S_{\mathrm{Irr}}\left(\lambda \right)\cdot \frac{\lambda }{1239.8}$$

(11) $$J_{\mathrm{SC}}=\int_{\lambda_{1}}^{\lambda_{2}}J_{\mathrm{SC}}\left(\lambda \right)\;d\lambda$$

The calculated J_SC_ provides a practical meaning for the IPCE(λ), which is often denoted as the external quantum efficiency (EQE). As the EQE is expressed by Equation (2), it is impossible to analyze the AL’s facility with the J_SC_ or IPCE specifically. For this reason, the IQE with Q_AL_ is estimated despite the time-consuming TMM treatment, including the estimation of all the required optical constants. It should be noted that there is an alternative measure to express the overall conversion efficiency of the device, namely the spectral response, SR(λ), as expressed in Equation (12). The SR(λ) with a typical A/W unit is beneficial because it does not require a specific P_in_. The SR(λ) spectrum for a 100 nm thick AL is shown in Figure 5 as (B) P_AL_.

(12) $$\mathrm{SR}\left(\lambda \right)=\mathrm{IPCE}\left(\lambda \right)\cdot \frac{\lambda \;\left(\mathrm{nm}\right)}{1239.8}$$

Figure 5 shows the calculated Q_AL_, P_AL_, and J_AL_ values for an AL with a thickness of 100 nm. In addition, the J_(2+3+5+6)_ values for all layers except the AL and incoherent glass substrates are shown. The scale of J_(2+3+5+6)_ was two-fold higher than that of J_AL_ because of its smaller amplitude. Despite the smaller amplitude of J_(2+3+5+6)_, there is a large amount of photon absorption that cannot contribute to the photogeneration of CCs in a device significantly. This must be an inadvertent loss from the viewpoint of the total incident light, as the loss is mainly caused by reflection and absorption by the glass substrate. For the device structure in this study, the total portion of parasite absorption caused by layers 2, 3, 5, and 6 was approximately 10% that of the AL. The relative portion of parasitic absorption is comparable to the difference between the EQE and IQE [8]. To prevent such a loss, the thicknesses of layers 2, 3, 5, and 6 must be controlled to suppress parasite absorption by attaining the PCE, which is often challenging because of the sensitive compromising nature of the device optimization process. Indeed, such considerations are only possible when the Q of each layer can be analyzed, for example, by using the TMM.

**Figure 6:**
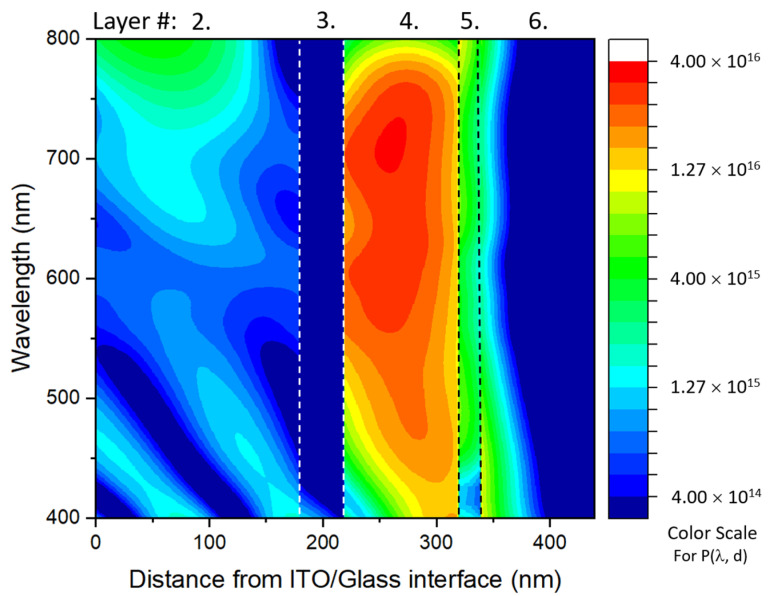
Three-dimensional resolved P(λ, d) map calculated using the TMM for an AL with a thickness of 100 nm. Layer # is shown at the top of the map in Figure 2.

As shown in Figure 5B, the P(λ) spectra can reflect the photon capturing ability as photon numbers because the photon numbers at a constant power increase with the increasing wavelength, which is more intuitive for evaluating the wavelength dependence of the photon capturing ability of a device. Therefore, a three-dimensional map of P(λ, d) is shown in Figure 6 to illustrate the solar power harvesting situation by capturing not only the spectrally resolved photon number, but also the spatially resolved photon number. Obviously, an AL has a maximum amplitude where the other layers only have a moderate absorption because subsidiary layers cannot usually contribute to the CC photogeneration. It is noteworthy that the color scale of the contour map is on a logarithmic scale to better show lower responses, although the area marked with the color scale from green to blue is practically meaningless for photovoltaic applications.

**Figure 7:**
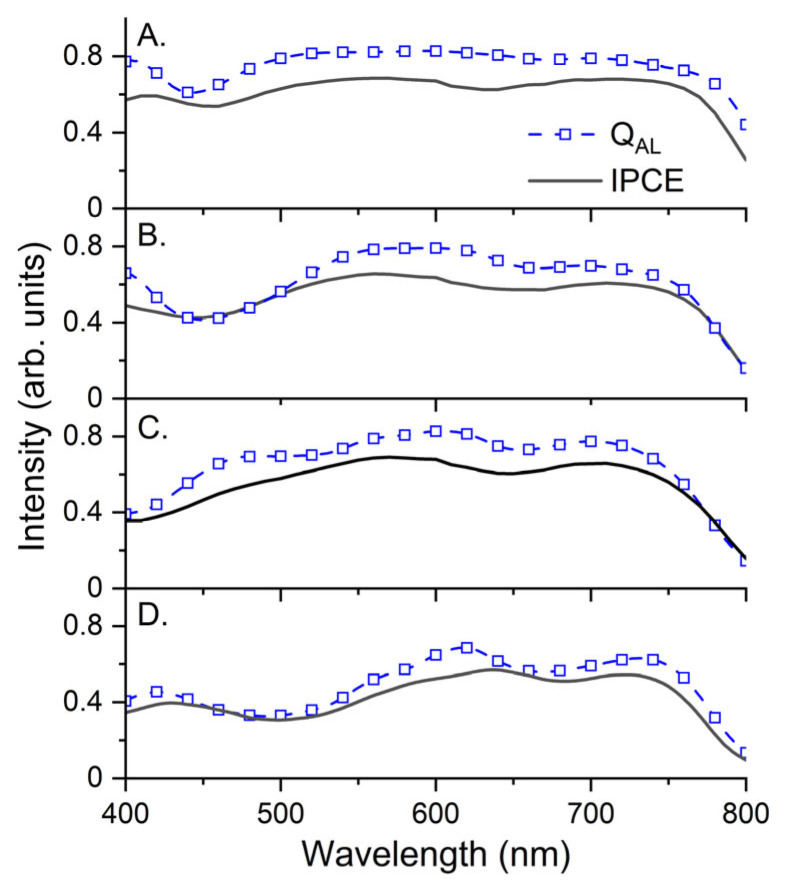
Experimental IPCE (solid lines) and calculated Q_AL_ spectra (dashed lines with open rectangles) via TMM for 350 nm (**A**), 200 nm (**B**), 100 nm (**C**), and 50 nm (**D**), respectively.

In Figure 7, the Q_AL_ is calculated via the TMM, and the experimentally estimated IPCE spectra of the devices with various AL thicknesses are shown. The Q_AL_ is obtained by the spatial integration of the Q_AL_ (λ), as expressed in Equation (7). The spectral shapes of the Q_AL_ and IPCE have a close similarity because CC photogeneration occurs mostly in an AL of a PSC. The amplitudes of the IPCEs were approximately 10% lower than those of the corresponding Q_AL_. The differences must be caused by the loss of captured photons in the AL to the extracted CCs and become enhanced with the increasing AL thickness, which will be discussed in more detail in the next section. It should be noted that the TMM results are reliable over a wide range of AL thicknesses, confirming the accuracy of the entire TMM procedure used in this study. In addition to the spectra in Figure 7, the P(λ) spectra of all layers are shown for verification in Figure S4 of the Supplementary Materials.

**Figure 8:**
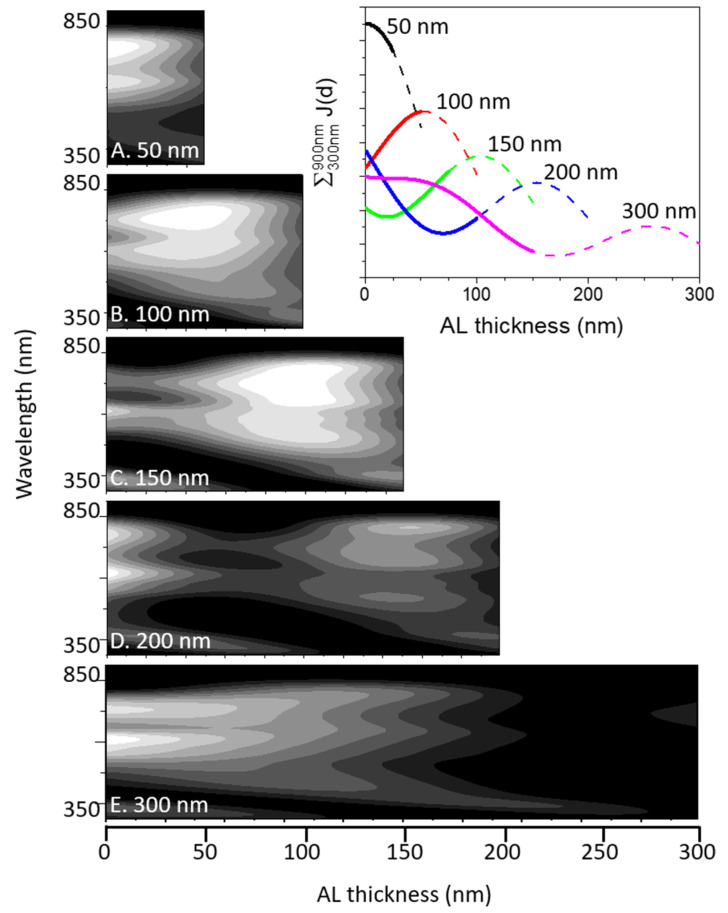
Three-dimensional maps of J_SC_ calculated via TMM as a function of AL thickness. Inset: J_AL_(d) integrated from 300 nm to 900 nm of the studied devices with AL thicknesses of 50 nm, 100 nm, 150 nm, 200 nm, and 300 nm. Front half part and rear half part are separately marked with thick solid lines and dashed lines, respectively.

In Figure 8, three-dimensional maps of the J_SC_ calculated using the TMM for various AL thicknesses are presented. The contour maps were plotted on a logarithmic gray gradient scale, and the darkest part was equivalent to 10% of the maximum amplitude. In the inset of Figure 8, the J_AL_(d) is spectrally integrated over the range of 300–900 nm for devices with AL thicknesses of 50, 100, 150, 200, and 300 nm. To clearly compare the spatial balancing, the front and rear halves of the plots are marked with thick solid lines and dashed lines, respectively.

As expected, the J map of the 300 nm AL case seem to mono-exponentially decrease with the increasing d, although the integrated J plot in the inset exhibits significant waviness caused by interference. However, the interference effect appearing in the 300 nm AL case seems to be the most moderate when compared to those with other AL thicknesses. In fact, the interference effect of the 200 nm AL case seems to be medium between the 150 and 300 nm thick AL cases. It is important to note that the shape of the J plot with a 100 nm thick AL seems to be the most symmetric among all the presented results. This observation can be compared with the J-V data, which reveal that the device with an approximately 100 nm thick AL has the maximum PCE. The J plot of the thin 50 nm thick AL case exhibits an extremely asymmetric shape, although this case has a relatively high IQE despite its low PCE owing to the weak absorption facility. Therefore, the CC generated within the thin AL can be effectively extracted from the device, even though its CC distribution is not spatially balanced.

## 3. Discussion

**Figure 9:**
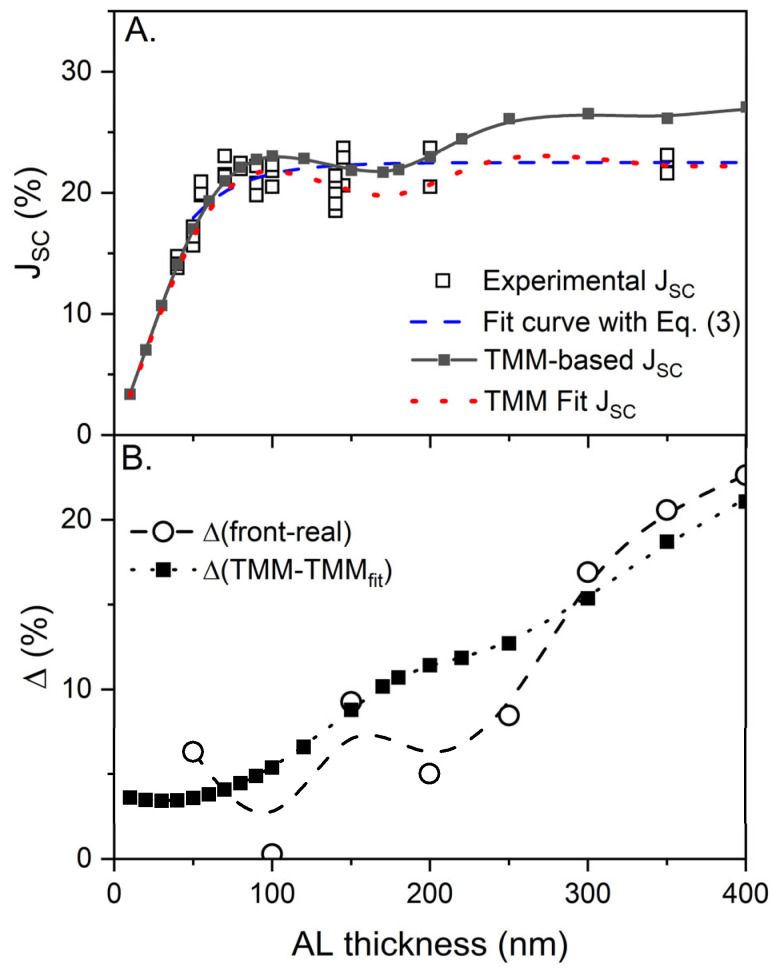
(**A**) A TMM-based loss-free J_SC_ plot (filled rectangles with solid guide line) and its fit curve (dot line) to the experimental J_SC_ (empty rectangles) and its mono-exponential fit curve (dashed line); (**B**) percent differences between the front half and the rear half of J plots shown in the inset of Figure 8 are plotted as AL thickness functions (open circles with dashed guide line), and percent differences between TMM-based loss-free J_SC_ and its fit data from (**A**) (filled rectangles with dotted guide line).

In Figure 9, the TMM-based ideal J_SC_ values are plotted as a function of the AL thickness. The TMM calculation for the ideal J_SC_ was performed assuming that the absorbed photons were converted to CCs and extracted to the external circuitry for detection without any loss. The ideal J_SC_ can serve as an upper limit of the maximum conversion efficiency after the initial stage of optical photon capture and exciton formation in a PSC device. Therefore, the ideal J_SC_ was compared to the experimental J_SC_ extracted from the corresponding J-V characteristics to reliably estimate the loss rate as a function of the AL thickness, as shown in Figure 9B.

The loss-free J_SC_ plot and mono-exponential fit curve to the experimental J_SC_ exhibited significant deviations. This implies that the model concept based on the incoherent optical condition possesses a significant mismatch with regard to the maximum achievable photon-capturing ability of an AL. Therefore, the accurate loss rate as a function of the AL thickness must be estimated using the TMM-based ideal J_SC_. Interestingly, only a single linear function of the CC loss was sufficient to fit the ideal J_SC_ with the experimental J_SC_, as shown in Figure 9A. The TMM fitting, including the interference feature, seems reasonable, and the obtained tendency is comparable to the result obtained using a fullerene-blended system [8,17].

Among the various loss mechanisms bound to the PSC operation, the spatial mismatching of the CC distribution appearing in the TMM-aided J(λ, d) maps in Figure 8 must be the central aspect of the AL- thickness-dependent loss. Undoubtedly, the harvested solar energy that must be extracted from a PSC is more likely to dissipate on-site when the AL thickness increases. By expanding the CC extraction path, the CCs must have a higher probability of colliding with moieties, resulting in energy scavenging or dissipation (referred to as loss mechanisms) via energy transfer or bimolecular recombination. Such an expansion of the CC extraction path can also be regarded as a delay of staying time before safe extraction.

To quantitatively evaluate the AL- thickness-dependent loss behavior in conjunction with the spatial distribution, the difference between the front half and rear half of the spectrally integrated J_AL_(d) are estimated, as shown in the inset of Figure 8, and the results are presented in Figure 9B. The difference between the front and rear parts, that is, the degree of spatial mismatch, has an impressively analogous functional dependence on the loss rate estimated using the TMM-aided J_SC_ values, as shown in Figure 9B. The spatial mismatch plot exhibits a wavy structure comparable to that of the J_SC_ results, as shown in Figure 9A. In addition, both the minimum of the spatial mismatching function and the maximum of the J_SC_ plots were located near an AL thickness of 100 nm. It can also be observed that the interference effect with an AL thickness greater than approximately 250 nm becomes significantly weaker, because most incident photons must be captured at the front part of the AL. Therefore, the two functions become closer, whereas those with thinner AL exhibit pronounced deviations, as indicated by the J maps in Figure 8.

Although a plausible correlation between the spatial balance of the CCs and AL- thickness-dependent loss probability could be demonstrated, a quantitative relation between the loss probability and the specific loss mechanisms must be further investigated. Nevertheless, the suggested concept of spatial CC balance can be used to disentangle the complicated fates of CCs as well as excitons not only for PSCs, but also for novel organic optoelectronics.

## 4. Materials and Methods

The experiments utilized two compounds, referred to as PBDB-T-2F and ITIC-4F [28,29,30]. The materials were purchased from 1-materials Inc. (Dorval, QC, Canada), and their chemical structures and full names are shown in the Supplementary Materials (Figure S1).

The UV-VIS spectra of the pristine and blended films with a 50:50 wt% ratio were spin-cast on quartz substrates. The film preparation conditions were kept consistent with those of the ALs in the photovoltaic devices to enable a reliable comparison between the spectroscopic and device properties. A two-beam UV-VIS spectrometer (Neosys-2000, Scinco, Seoul, Republic of Korea) and fiber-coupled spectrometer (AvaSpec-ULS2048, Avantes, Apeldoorn, The Netherlands) were used to obtain the absorptance, reflectance, and transmittance spectra of the film samples. Additional reflectance spectra were measured for these devices because of their opaque metallic electrodes.

For device preparation, a zinc oxide (ZnO) thin layer and blended AL were successively spin-cast onto indium tin oxide (ITO)-covered glass substrates, as described in a previous study [16], and molybdenum oxide/silver (MoO_3_/Ag) counter-electrodes were thermally evaporated. The device structure was glass (1 mm)/ITO (180 nm)/ZnO (40 nm)/AL (d nm)/MoO_3_ (20 nm)/Ag (100 nm). For the AL- thickness-dependent experiments, the AL thickness was varied from approximately 40 to 350 nm by changing the number of revolutions per minute (RPM) of the spin coater. To obtain AL thicknesses of less than approximately 100 nm, a low-concentration (5 mg/mL) solution was used, whereas devices with AL thicknesses greater than approximately 100 nm were coated with a high-concentration (10 mg/mL) solution. A plot of AL thickness versus RPM is presented in Figure S1 of the Supplementary Materials. The results reveal that the solution concentrations in our case did not seriously affect the continuity of the device properties obtained in this study, although the processing conditions may sensitively affect the resulting device properties, as reported by Gupta et al. [31,32].

The AL thickness was measured using a surface profiler (XP-200, AMBiOS, Totnes, UK). All devices were characterized to obtain their device parameters, that is, V_OC_, J_SC_, FF, and PCE, using J-V measurements under standard illumination conditions of 1-sun, referred to as Air Mass 1.5 G spectral irradiance, at a power of 1 kW/m^2^ [24,33]. The IPCE and IQE were also estimated using an XE-7 (PV Measurement Inc., Boulder, CO, USA), as described in detail elsewhere [11].

To perform TMM, the optical constants, refractive indices (n), and extinction coefficients (k) of all layers used in this study were estimated using a variable-angle spectroscopic ellipsometer (VASE, J. A. Woollam Co., Inc., Lincoln, NE, USA). For the practical calculation of various TMM-based spectra, we used a home-made program with the stable public domain program “OpenFilter” [34], especially for cross- checking purposes. It is noteworthy that the core part of the TMM calculation, the basic matrix formalism used to deal with the optical electric field inside devices, is not described in this study because it is well established; therefore, we want to reduce space-consuming repetition of the existing literature [11]. However, calculation methods to obtain specific spectra leading to crucial conclusions, including verification for calculation in this study, are described in detail along with equations and figures in Section 2.

## 5. Conclusions

To understand the AL- thickness-dependent photovoltaic nature of PSCs, a well-known NFA-blended polymer BHJ system, consisting of ITIC-4F and PBDBT-2F, was chosen for the investigation. PSCs with AL thicknesses ranging from 50 to 350 nm were prepared with the following multilayer structure: glass/ITO/ZnO/AL/MoO_3_/Ag. The estimated device parameters of the J_SC_, FF, and PCE were dependent on the AL thickness. The J_SC_ as a function of the AL thickness exhibited a gradual convergence, which is expected under both coherent and incoherent optical conditions. However, the J_SC_ plot revealed pronounced interference fluctuations, which necessitated the inclusion of interference considerations to analyze the detailed photon absorption aspects of PSCs.

The internal absorption, Q, of each layer in the PSCs is important not only to analyze the initial photon capture by an AL in devices, but also to verify the degree of parasitic absorption by subsidiary layers, which is an important hindrance to achieving a high PCE. To estimate the precise Q spectra of each layer, calculations using the TMM with experimental optical constants were performed. The AL- thickness-dependent spectrally and spatially resolved J_AL_(λ, d) maps revealed that the Q of the NFA-blended PSCs increased with the increasing AL thickness, whereas their PCE decreased with the increasing AL thickness after reaching a maximum near 100 nm. The TMM-aided loss-free ideal J_SC_ could be successfully fitted to the experimental J_SC_ using a single linear loss function; thus, an accurate thickness-dependent loss probability could be estimated.

Furthermore, the TMM results were evaluated to analyze the AL- thickness-dependent spatial distribution of the photogenerated CCs in conjunction with the thickness-dependent loss probability. The results reveal that the AL of the 100 nm thick device had the most homogeneous CC distribution, whereas the other cases did not exhibit a comparable homogeneous distribution. The devices with an AL thickness greater than ~250 nm lose their interference effect significantly because photon capture occurs mainly in the front part of thicker ALs. The AL-thickness-dependent loss function is comparable to the AL-thickness-dependent spatial mismatch function proposed in this study. Accordingly, a possible model to explain the deviation between steadily increasing the Q and increasing the loss probability can be suggested in terms of the spatial balancing of the photogenerated CC distribution within the ALs of PSCs.

## Figures and Tables

**Table 1:** Overview of photovoltaic device parameters.

AL Thickness (nm)	FF (%)	V_OC_ (V)	J_SC_ (mA/cm^2^)	PCE (%)
50	62.5	0.85	17.2	9.1
80	60.4	0.84	21.4	10.8
100	57.3	0.80	22.3	10.0
140	60.4	0.81	19.1	9.2
200	42.1	0.80	23.7	8.0
350	35.1	0.81	23.1	6.6

## Data Availability

The data presented in this study are available upon request from the corresponding authors.

## Supplementary Materials

The following are available online at https://www.mdpi.com/article/10.3390/molecules28155823/s1, Figure S1: Chemical structures of (A) 3,9-bis(2-methylene-((3-(1,1-dicyanomethylene)-6,7-difluoro)-indanone))-5,5,11,11-tetrakis(4-hexylphenyl)-dithieno [2,3-d:2′,3′-d′]-s-indaceno[1,2-b:5,6-b’]dithiophene (ITIC-4F), and (B) poly[(2,6-(4,8-bis(5-(2-ethylhexyl-3-fluoro)thiophen-2-yl)-benzo[1,2-b:4,5-b′]dithiophene))-alt-(5,5-(1′,3′-di-2-thienyl-5′,7′-bis(2-ethylhexyl)benzo[1′,2′-c:4′,5′-c′]dithiophene-4,8-dione)] (PBDB-T-2F) (R_1_ = 2-ethylhexyl, R_2_ = hexyl).; Figure S2: AL thickness as a function of spin-coating RPM; Figure S3: Transmittance and reflectance of the glass substrate used in this study; Figure S4: P spectra of all layers except incoherent glass substrate of a device with a thickness of 100 nm are shown.
