# Supplementary material for: Spatial Balance of Photogenerated Charge Carriers in Active Layers of Polymer Solar Cells
Source: Molecules. 2023 Aug 2;28(15):5823. doi: 10.3390/molecules28155823 (PMC10421118; doi:10.3390/molecules28155823)
Supplement: Supplementary file 1 [file molecules-28-05823-s001.zip › molecules-2512344-supplementary.pdf]

molecules

Supplementary Material

Spatial Balance of Photogenerated Charge Carriers in Active layers of Polymer Solar Cells

Chan Im[1, *], Sang Woong Kang[1], Jeong Yoon Choi[1], Jongdeok An[1], Júlia Mičová[2] and Zdeněk Remeš[3].

[1] Department of Chemistry, Konkuk University, 120 Neungdong-ro, Gwangjin-gu, Seoul 05029, South Korea

[2] Institute of Chemistry, Slovak Academy of Sciences, Dúbravská Cesta 9, SK-845 38 Bratislava, Slovakia

[3] Institute of Physics, Czech Academy of Sciences, Na Slovance 1999/2, 182 21 Prague 8, Czech Republic

* Corresponding author: chanim@konkuk.ac.kr

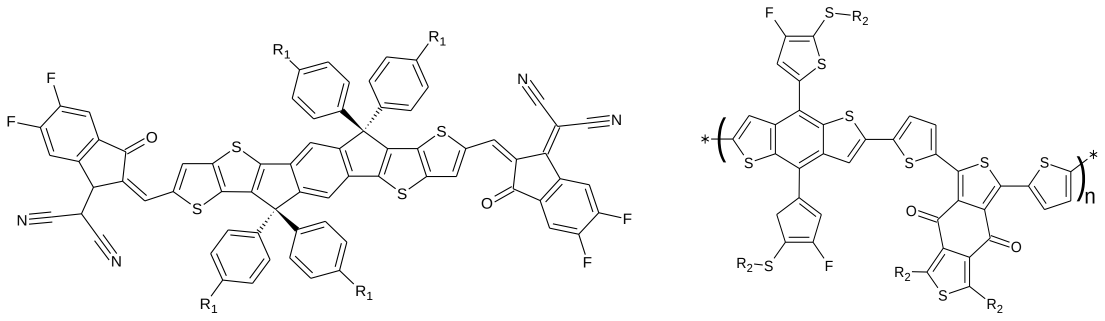

(A) ITIC-4F (B) PBDB-T-2F

Figure S1. Chemical structures of (A) 3,9-bis(2-methylene-((3-(1,1-dicyanomethylene)-6,7-difluoro)-indanone))-5,5,11,11-tetrakis(4-hexylphenyl)-dithieno[2,3-d:2’,3’-d’]-s-indaceno[1,2-b:5,6-b’]dithiophene (ITIC-4F), and (B) poly[(2,6-(4,8-bis(5-(2-ethylhexyl-3-fluoro)thiophen-2-yl)-benzo[1,2-b:4,5-b’]dithiophene))-alt-(5,5-(1’,3’-di-2-thienyl-5’,7’-bis(2-ethylhexyl)benzo[1’,2’-c:4’,5’-c’]dithiophene-4,8-dione)] (PBDB-T-2F). (R_1 = 2-ethylhexyl, R_2 = hexyl).

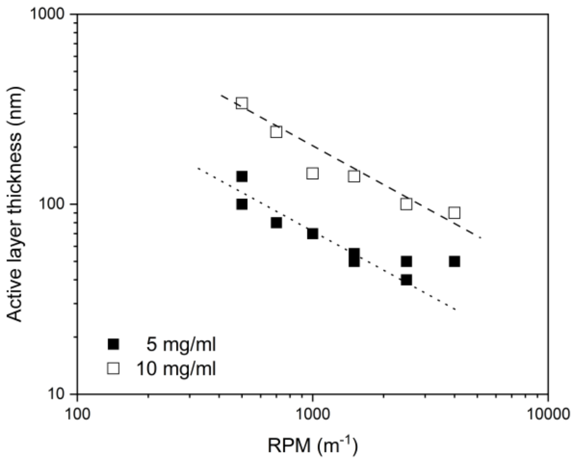

Figure S2. AL thickness as a function of spin-coating RPM.

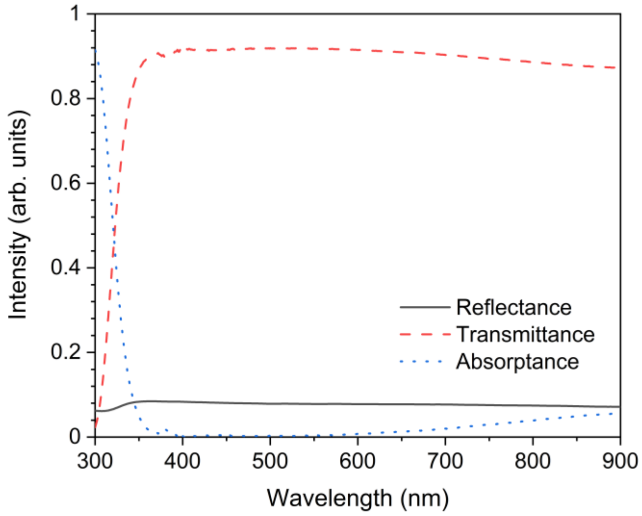

Figure S3. Transmittance and reflectance of glass substrate used in this study.

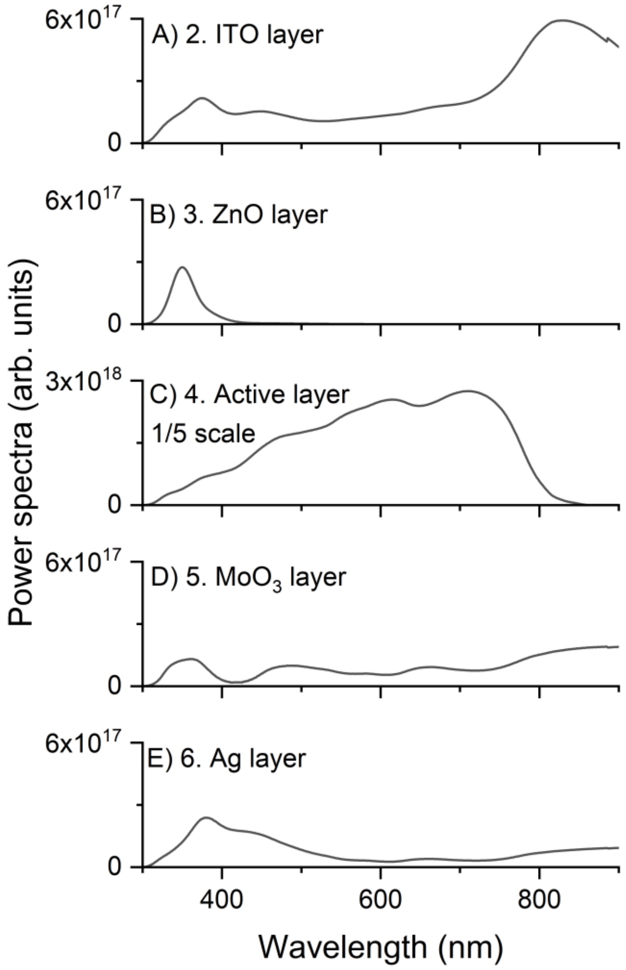

Figure S4. The P spectra of all the layers, except the incoherent glass substrate of a device with a thickness of 100 nm, are shown. Owing to the significant amplitude difference, a 1/5 scale was used only for the active layer.
